# “Dancing Eye Syndrome” Secondary to Opsoclonus-Myoclonus Syndrome in Small-Cell Lung Cancer

**DOI:** 10.1155/2014/545490

**Published:** 2014-03-23

**Authors:** S. Laroumagne, Xavier Elharrar, B. Coiffard, J. Plojoux, H. Dutau, D. Breen, P. Astoul

**Affiliations:** ^1^Department of Thoracic Oncology, Pleural Diseases, and Interventional Pulmonology, AP-HM-Hôpital Nord, Chemin des Bourrely, 13326 Marseille Cedex 20, France; ^2^Aix-Marseille University, 13005 Marseille, France; ^3^Department of Respiratory Medicine, University Hospitals Galway, Galway, Ireland

## Abstract

Among paraneoplastic neurologic disorders (PND), opsoclonus-myoclonus syndrome, so-called “dancing eye syndrome,” is a rare disorder combining multivectorial eye movements, involuntary multifocal myoclonus, and cerebellar ataxia. Although several paraneoplastic antibodies against postsynaptic or cell-surface antigens have been reported, usually most patients are serum antibody negative. We report a 65-year-old patient with opsoclonus-myoclonus syndrome revealing a small-cell lung carcinoma. If serologic antineuronal anti-body screening was negative, autoantibodies against glutamic acid decarboxylase (anti-GAD) were positive. Despite the specific anticancer treatment and high dose corticosteroids, the patient developed a severe and progressive encephalopathy and died 10 days later.

## 1. Introduction

Opsoclonus-myoclonus syndrome (OMS) is characterized by rapid, involuntary multivectorial eye movements without intersaccadic intervals. It is associated with involuntary multifocal myoclonus mainly affecting the trunk, limbs, and head. Cerebellar ataxia and uncoordinated voluntary movements may also be seen. Pathophysiologically OMS appears to be an immune-mediated disease where positive titres directed at anti-neuronal antibodies in the serum and cerebrospinal fluid (CSF) are occasionally detected. OMS has been linked to infections and metabolic disorders and has been documented as a paraneoplastic syndrome.

The paraneoplastic opsoclonus-myoclonus syndrome, also called “dancing eyes syndrome,” is a rare entity among the paraneoplastic neurologic disorders (PND) [1]. OMS is mainly associated with neuroblastoma in children; however, it has also been reported in patients with small-cell lung cancer (SCLC) and breast and ovarian malignancies. In addition, there have been case reports in the literature which have associated OMS with non-small-cell lung cancer, melanoma, sarcoma, and non-Hodgkin's lymphoma [2–5].

In this paper, we report the case of a male patient with pathologically proven small-cell lung cancer who presented with symptoms compatible with OMS. This was the first clinical manifestation of an underlying malignancy. He demonstrated a rapid deterioration in his clinical state leading to death despite the introduction of chemotherapy active against the small-cell lung cancer.

## 2. Case Report

A 65-year-old man presented to the emergency department of our institution with a history of left hemithoracic pain, weight loss, gait ataxia, and tremor. He had a 120-pack-year smoking history and known ischemic cardiomyopathy with previous coronary stenting.

**Figure 1:**
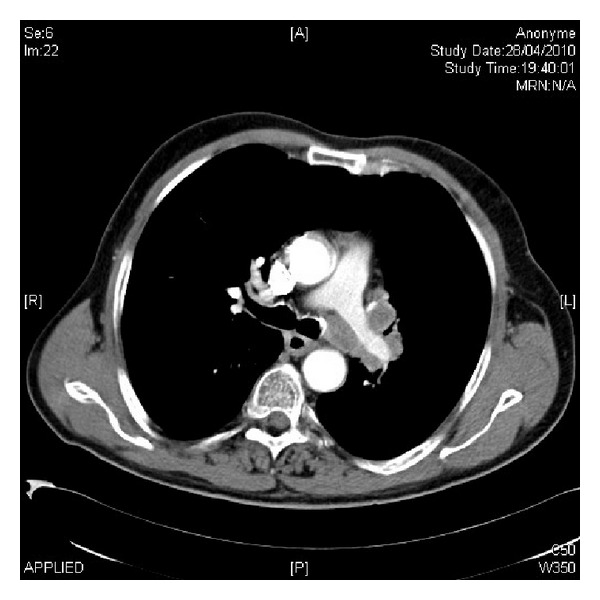
Thoracic imaging by computed tomography (CT) revealed a left hilar mass with associated left hilar and mediastinal lymphadenopathy.

On physical examination he had a reduced performance status (PS 1, ECOG classification) and neurological assessment revealed tremor, gait ataxia, and mild involuntary limb jerking which resulted in the patient remaining bedbound despite any evidence of cognitive impairment and sensory or motor deficit. The initial chest X-ray demonstrated an enlarged left hilum. Thoracic imaging by computed tomography (CT) revealed a left hilar mass measuring 26 mm with associated left hilar and mediastinal lymphadenopathy (>1 cm short axis) (Figure 1). Endobronchial ultrasound (EBUS) and transbronchial needle aspiration (TBNA) of the involved lymph nodes were performed and this confirmed a diagnosis of small-cell lung cancer (SCLC). The diagnostic workup was completed with 18-fluorodeoxyglucose-positron emission tomography (18-FDG-PET) which staged the SCLC as limited disease.

**Figure 2:**
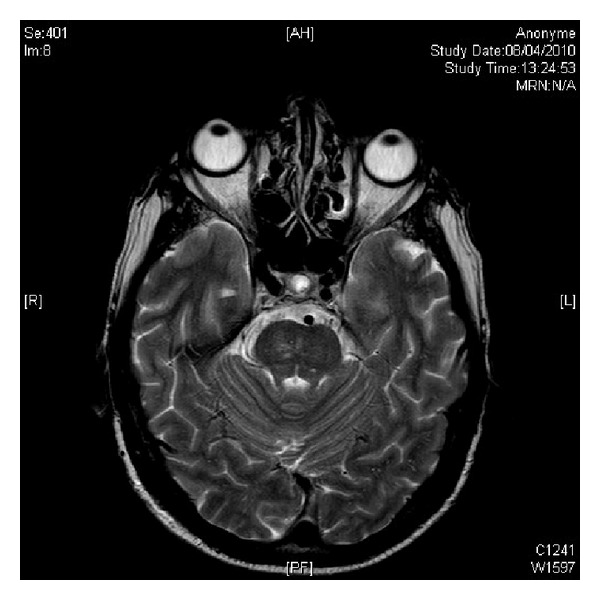
Magnetic resonance imaging (MRI) of the brain without ischemic changes, metastatic lesions, or abnormal enhancement with gadolinium.

**Figure 3:**
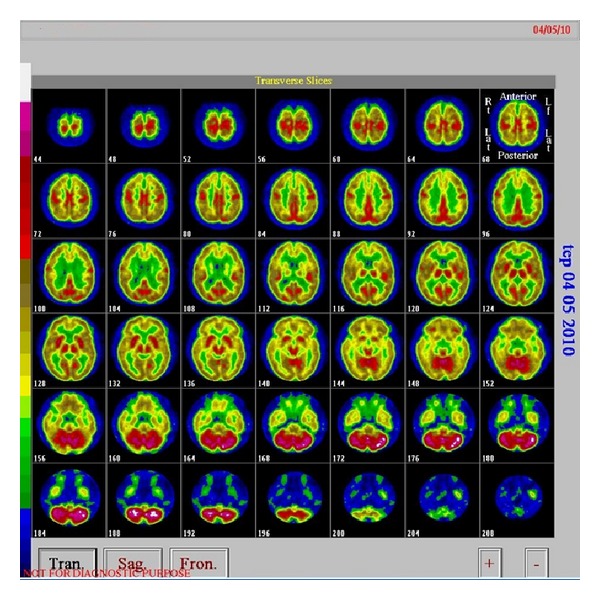
Brain 18-FDG-PET showing diffuse cerebral hypometabolism with cerebellar hypermetabolism (usually reported finding in PND).

Further workup for a potential cerebellar paraneoplastic disorder revealed a normal electroencephalography, normal cell counts, protein levels, viral markers, and negative cytology in the cerebrospinal fluid (CSF) analysis. Magnetic resonance imaging (MRI) of the brain did not reveal ischemic changes, metastatic lesions, or abnormal enhancement with gadolinium (Figure 2). Brain 18-FDG-PET demonstrated diffuse cerebral hypometabolism with cerebellar hypermetabolism which is a reported finding in PND (Figure 3).

As the index of suspicion for PND remained high, we performed serologic anti-neuronal antibody screening. Anti-Hu, Ant-Ri, anti-Yo, anti-CV2, anti-Ma1, anti-Ma2, anti-amphiphysin, and anti-VGKC were all negative. A serum glutamic acid decarboxylase antibody radio immunoassay (GAD65 Ab Assay—AntiGAD Antibodies blood test) was positive (1.1 U/mL) (Titers generally < or = 0.03 U/L).

Chemotherapy was commenced using the combination of carboplatinum and etoposide with intravenous corticosteroids. Cisplatinum administration was contraindicated because of the recent history of ischemic cardiomyopathy and a measured ventricular ejection fraction at 40%. Despite the initiation of specific treatment of the underlying malignancy, dramatic neurologic deterioration occurred and the patient developed multivectorial saccadic eye movements with associated massive myoclonic head, trunk, and limb movements. This deterioration resulted in a comatose state necessitating endotracheal intubation, sedation, and transfer to the intensive care unit.

The patient died 10 days later due to severe and progressive encephalopathy despite the specific anticancer treatment and high dose corticosteroids.

## 3. Discussion

Paraneoplastic OMS has been associated with SCLC and breast and ovarian malignancies in adults and antineuronal antibodies have been occasionally detected [6, 7]. Among the anti-neuronal antibodies, anti-RI, a nonsurface antigen type, Zic2 antigen (anti-Zic2), and more recently adenomatous polyposis coli antigen (anti-APC) are the most frequently detected [7, 8]. The enzyme called glutamic acid decarboxylase is responsible for converting glutamic acid to GABA which is found in the cerebellum. For some authors cerebellar ataxia and other cerebellar disorders should be the result of a lack of GABA which is targeted by anti-GAD antibodies. Patients with cerebellar ataxia of an unknown cause should have an anti-GAD test [8]. Nevertheless, the majority of patients with PND and OMS are antibody negative; however, this does not exclude the diagnosis.

OMS is an involuntary multidirectional eye movement syndrome (spontaneous conjugate saccades in all directions of gaze) accompanied by myoclonic jerks (irregular muscle spasms of the head, trunk, or extremities). Cerebellar ataxia (POMA syndrome), tremor, and encephalopathy are common. As with the majority of PND, OMS precedes the diagnosis of cancer (in up to two-thirds of cases), but it can also be detected during the follow-up period suggesting recurrent disease [9]. The pathophysiological mechanism is still unclear. Some researchers suggest disinhibition of cerebellar nuclei or dysfunction of premotor neurons of the brainstem [10, 11].

In our case the patient initially presented with cerebellar symptoms. Investigations were consistent with a cerebellar paraneoplastic disorder supported by a histologically proven diagnosis of SCLC, brain 18-FDG-PET findings, and positive anti-GAD antibodies [12, 13]. Our patient showed hypermetabolism in the cerebellum on FDG-PET scan. The hypermetabolism should be related to inflammatory changes as previously shown in the literature when stereotaxic biopsy is done revealing lymphocytic infiltrations and reactive gliosis [14] or at autopsy studies with severe loss of Purkinje cells in combination with various degrees of inflammatory [15]. Conversely studies performed more than 1year after the onset of symptoms consistently show absence of inflammatory infiltrates (hypometabolism at FDG-PET) suggesting the regression of inflammatory process [16].

The rapid progression towards OMS with multidirectional eye movements and jerking body movements and ultimately coma strongly suggests a paraneoplastic phenomenon, even if standard serological screening for anti-neuronal antibodies associated with OMS remained negative. It is therefore not surprising that current recommendations are to repeat the screening tests every six months if malignancy is not initially discovered [17]. However, reports in the literature suggest that up to 50% of patients have improvement in their neurologic function and the prognosis in small-cell lung cancer patients is better if associated with positive neuronal antibodies tests [18].

In summary we believe that new approaches to detect anti-neuronal antibodies will allow faster detection of the underlying malignancy of PND leading to an earlier treatment of the disease with better outcomes for the patients.
